# A simplified feline phantom and Monte-Carlo simulation can be used to evaluate radiation exposure to veterinary staff during [^211^At]NaAt treatment

**DOI:** 10.3389/fvets.2026.1897774

**Published:** 2026-08-07

**Authors:** Tetsuya Sakashita, Kota Onuma, Akito Sugasawa, Ichiro Sasaki, Fuga Sasaki, Hiromitsu Daisaki, Noriko S. Ishioka, Nobuhiko Ito, Takehiko Kakizaki

**Affiliations:** 1 Department of Quantum-Applied Biosciences, National Institutes for Quantum Science and Technology, Takasaki, Gunma, Japan; 2 Department of Radiological Technology, Gunma Prefectural College of Health Sciences, Maebashi, Gunma, Japan; 3 School of Veterinary Medicine, Kitasato University, Towada, Aomori, Japan; 4 Research Center for Accelerator and Radioisotope Science, Tohoku University, Sendai, Miyagi, Japan

**Keywords:** [^211^At]-labeled radiopharmaceuticals, 3D-printed phantom, feline thyroid disease, Monte Carlo simulation, veterinary nuclear medicine, veterinary occupational exposure

## Abstract

**Introduction:**

Targeted alpha therapy using ^211^At offers high therapeutic efficacy with relatively low occupational radiation exposure. However, in veterinary medicine, staff exposure—particularly during close animal handling—remains insufficiently evaluated. We quantitatively evaluated external occupational radiation exposure to veterinary staff during simulated clinical use of ^211^At-labeled radiopharmaceuticals, utilizing dose measurements and numerical simulations based on a feline phantom model.

**Materials and methods:**

A spherical [^211^At]NaAt source was placed in the neck of a CT-derived, 3D-printed feline phantom. Ambient dose equivalent rates were measured at two to five distances ranging from the phantom surface to 1 m using a NaI(Tl) scintillation survey meter, with measurement geometry reproducibly controlled by a dedicated 3D-printed phantom. Concurrently, Monte Carlo simulations using the Particle and Heavy Ion Transport Code System (PHITS) modeled ^211^At photon transport to calculate ambient dose equivalent rates. Simulation results were compared with experimental measurements to validate dosimetric evaluations.

**Results:**

Measurements of the ambient dose equivalent rate, Ḣ^*^(10), using a spherical source and simplified 3D-printed feline phantom showed good agreement with PHITS Monte Carlo simulations at distances within 1 m, except at very low dose rates near the detection limit. Simplified analytical calculations (FORM) consistently overestimated dose rates, particularly at distances within 10 cm, by up to two orders of magnitude, whereas PHITS provided conservative yet realistic estimates. Gamma-ray spectroscopy demonstrated that photons emitted from ^211^At and its progeny were detectable only at close distances (~10 cm), suggesting that local shielding may be necessary when high activities are administered. In a simplified clinical scenario, the estimated occupational radiation exposure per [^211^At]NaAt treatment was low, with effective dose, skin equivalent dose, and eye lens equivalent dose values not exceeding 1.11 μSv for any veterinary staff category.

**Conclusion:**

The combined use of a 3D-printed phantom and numerical simulations demonstrated that occupational doses associated with [^211^At]NaAt treatment were within acceptable levels, and well below the occupational dose limits recommended by the ICRP, supporting the radiological safety of the procedure. The framework is readily applicable to other organs or animal species, thereby supporting the safe and feasible expansion of veterinary nuclear medicine.

## 1 Introduction

Halogen radionuclides, such as ^1^^3^^1^I (T_1/2_ = 8.0 d) and ^2^^1^^1^At (T_1/2_ = 7.2 h), are effective for treating thyroid diseases. In human nuclear medicine, the use of [^1^^3^^1^I]NaI for diagnosing and treating thyroid diseases has been established and widely adopted for more than half a century (2). In recent years, advances in the clinical application of targeted radionuclide therapy (TRT) using [^2^^1^^1^At]NaAt have led to lower radiation exposure to medical staff (3) and high therapeutic efficacy (3, 4). By contrast, in veterinary medicine, the use of [^1^^3^^1^I]NaI is widely adopted for managing feline thyroid diseases (5). However, in several countries including Japan, the use of therapeutic radionuclides (radiopharmaceuticals) in veterinary medicine is restricted by regulatory and radiation-protection requirements, including the lack of approved therapeutic radiopharmaceuticals for veterinary use and the lack of established safety guidelines for managing radiation exposure to veterinary staff, animal owners, and members of the public. Therefore, neither [^1^^3^^1^I]NaI nor [^2^^1^^1^At]NaAt can currently be introduced into veterinary clinical practice. To address this issue, it is essential to scientifically evaluate radiation exposure to veterinary medical staff and to establish objective safety criteria. Although external dose assessments of ^2^^1^^1^At sources have previously been conducted and their results have been consolidated into effective dose rate constants for radiation protection purposes (3), no studies have evaluated radiation exposure under conditions representative of veterinary treatment, in which a ^2^^1^^1^At-labeled radiopharmaceutical is administered to an animal. Therefore, we focused on assessing the occupational radiation exposure of veterinary staff associated with [^2^^1^^1^At]NaAt treatment.

The assessment of radiation exposure in veterinary radionuclide therapy (veterinary TRT) involves challenges that are not fully addressed by current radiation-protection practices in human nuclear medicine. In human TRT, dose rates measured at a distance of 1 m from the patient are commonly used for patient-release criteria and for estimating radiation exposure to caregivers and members of the public (3). However, veterinary medical staff are frequently required, as part of their routine clinical duties, to work at distances substantially shorter than 1 m when handling or examining animals. Furthermore, the effective dose rate, **Ė**, cannot be measured directly in routine practice. Therefore, assessment of radiation exposure in veterinary TRT generally requires dose-rate measurements obtained with a survey meter in addition to the effective dose rate, **Ė**. The quantity measured with a survey meter is the operational quantity, Ḣ^*^(10), which is defined by the International Commission on Radiation Units and Measurements (ICRU) and recommended by the International Commission on Radiological Protection (ICRP). In this notation, the asterisk denotes an ambient operational quantity for area monitoring, and the 10 indicates a reference depth of 10 mm in a tissue-equivalent sphere for assessing strongly penetrating radiation. For these reasons, the present study evaluated ambient dose equivalent rates not only at 1 m but also at shorter distances representative of veterinary clinical procedures. In general, the protection quantity, **Ė**, which is used for radiation protection, is smaller than the operational quantity, Ḣ^*^(10), which is applied in practical measurements. In Japan, measurements are based on Ḣ^*^(10) in practice, whereas radiation protection regulations primarily adopt **Ė**, with Ḣ^*^(10) being used in cases in which the application of **Ė** is difficult. Veterinary clinical practice differs from human medical practice in that companion animals often require physical restraint, close handling, or cage management because verbal communication is not possible. Therefore, external radiation exposure to veterinary staff at distances of less than 1 m should be considered when establishing radiation-protection procedures (6, 7). However, to date, no reports have evaluated survey meter measurements or radiation doses associated with [^211^At]NaAt at such short distances in veterinary settings.

One possible approach to addressing this issue is the use of three dimensional (3D)-printed physical phantoms in combination with numerical simulations. A direct method to resolve this problem would be to administer [^2^^1^^1^At]NaAt to feline patients with thyroid disease and to measure radiation doses at the body surface and at close distances. However, the conduct of animal experiments has been increasingly restricted worldwide (8). In addition, in Japan, the use of [^2^^1^^1^At]NaAt in veterinary medicine is not permitted under current regulatory frameworks. Regarding assessment of radiation exposure to veterinary medical staff, a previous study in dogs evaluated ambient dose distributions during diagnostic procedures using [^18^F]fluoro-deoxy-d-glucose positron emission tomography (^1^8F-FDG PET) radiopharmaceuticals based on numerical simulations with simple-shaped phantoms and reported good agreement between simulated and measured values (7). In human nuclear medicine, assessments of radiation exposure to medical staff and others have been conducted using water phantoms and computational phantoms (9, 10). More recently, 3D-printed phantoms have been increasingly used in various fields, including medical imaging and radiation dose assessment (1).

Given the above background, the aim of this study was to develop a methodology for evaluating external radiation exposure to veterinary medical staff during clinical practice by using 3D-printed physical phantoms and numerical simulations, with a view toward the future introduction of the ^2^^1^^1^At-labeled radiopharmaceutical, [^2^^1^^1^At]NaAt, into veterinary medicine. The objectives of this study were threefold. First, the ambient dose equivalent rate at the body surface and at distances less than 1 m was measured using a 3D-printed simplified feline phantom containing spherical inserts filled with [^2^^1^^1^At]NaAt, and spectral measurements were performed to assess the necessity of lead shielding at close distances. Second, a numerical simulation methodology capable of reproducing the measured results was established. Third, external radiation exposure to veterinary medical staff was evaluated under a simplified clinical scenario involving [^2^^1^^1^At]NaAt.

## 2 Materials and Methods

### 2.1 Preparation of [^211^At]NaAt and treatment

^211^At was produced at the Takasaki Ion Accelerators for Advanced Radiation Application (TIARA) facility of National Institutes for Quantum Science and Technology (QST) Takasaki through the ^209^Bi(^4^He, 2n)^211^At nuclear reaction using an azimuthally varying field cyclotron. ^211^At was separated from the irradiated bismuth targets and was purified using dry distillation. Subsequently, ^211^At was collected in a solution containing 1% ascorbic acid and 2.3% sodium hydrogen carbonate in accordance with a previously reported method (11). [^211^At]NaAt exhibits lower volatility than free ^211^At and thus reduces the risk of contamination incidents. The radioactivity concentration of the [^211^At]NaAt solution was determined by depositing 10 μL of the solution onto a thin circular filter paper disk that was 6 mm in diameter (Tokyo Roshi Kaisha, Tokyo, Japan), sealing the filter paper disk with transparent adhesive tape, and measuring the sample using a high-purity germanium (HPGe) detector (GEM15P4-70; SEIKO EG&G, Tokyo, Japan). In addition, 0.45 ml of the [^211^At]NaAt solution was loaded into a spherical insert with a maximum capacity of 0.5 mL (Hollow Spheres; DataSpectrum, Durham, NC). The administered activity of [^2^^1^^1^At]NaAt was set to 10–20 MBq, based on the upper limit of 10 MBq/kg used for dose design in human phase I clinical trials (3), an average body weight of 4.2 kg derived from 100 felines aged 7–15 years that were assumed to have thyroid disease, and an assumed thyroid uptake fraction of 30%. The ambient dose equivalent rate was measured in μSv/h using a NaI(Tl) scintillation survey meter TCS-1172 (ALOKA, Tokyo, Japan). In addition, to measure Ḣ^*^(10) at the body surface (at a distance of 2 cm corresponding to the reference position of the NaI(Tl) scintillation survey meter, TCS-1172), the administered activity of [^2^^1^^1^At]NaAt was set to approximately 5 MBq (4.7 MBq in the experiment), taking into account the maximum measurable dose rate of 30 μSv/h for the TCS-1172 surveymeter.

### 2.2 3D-printed jig and simplified feline phantom

**Figure 1:**
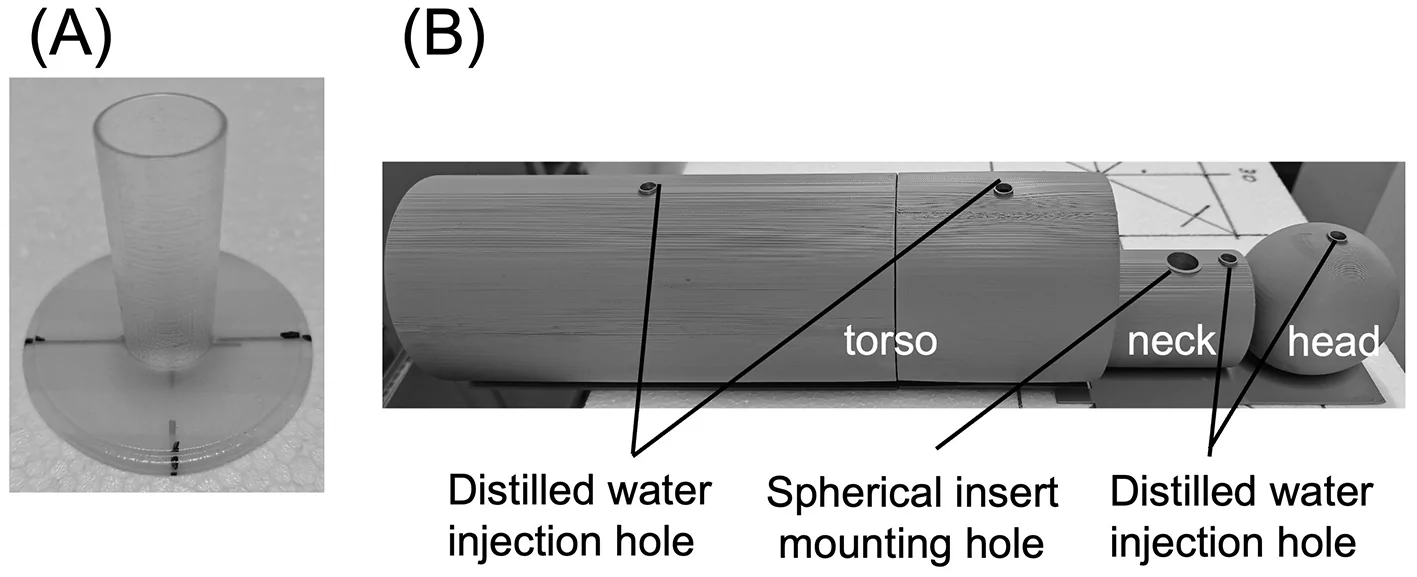
Three dimensional (3D)-printed jig **(A)** and simplified feline phantom (**B**). The spherical insert was enclosed in double vinyl bags and fixed to a 3D-printed jig. Marks were made on the auxiliary plate of the 3D-printed jig so that the position of the source center was consistently reproduced. The simplified feline phantom was constructed by connecting a head, neck, and torso with an overall length of 50.3 cm and a uniform thickness of 2 mm for each part. Each component included a distilled water injection hole or spherical insert mounting hole.

To evaluate and validate the performance of the Monte Carlo simulation, two dose rate measurement experiments were conducted using a spherical insert fixed to a jig and a 3D-printed simplified feline phantom. A 3D-printed jig was fabricated using an Agilista printer (KEYENCE Corp., Osaka, Japan). The spherical insert was enclosed in double vinyl bags to prevent radioactive contamination and fixed to a 3D-printed jig (Figure 1A). A simplified feline phantom (Figure 1B) was fabricated using a Bambu Lab H2D printer (Bambu Lab, Shenzhen, China). It was constructed by connecting a head (a sphere with a radius of 4.1 cm), neck (a cylinder with a radius of 3.2 cm and length of 7.5 cm), and torso (a cylinder with a radius of 5.5 cm and length of 34.6 cm). The external dimensions of the simplified feline phantom were determined based on body size measurements obtained from the computed tomography (CT, Activion 16, Canon Medical Systems, Japan) data of 10 feline clinical cases at the Veterinary Teaching Hospital, School of Veterinary Medicine, Kitasato University. A spherical insert including [^2^^1^^1^At]NaAt was positioned in the neck region to correspond to the anatomical location of the thyroid gland, while the remaining internal volume of the phantom was filled with distilled water.

### 2.3 Measurement geometry

**Figure 2:**
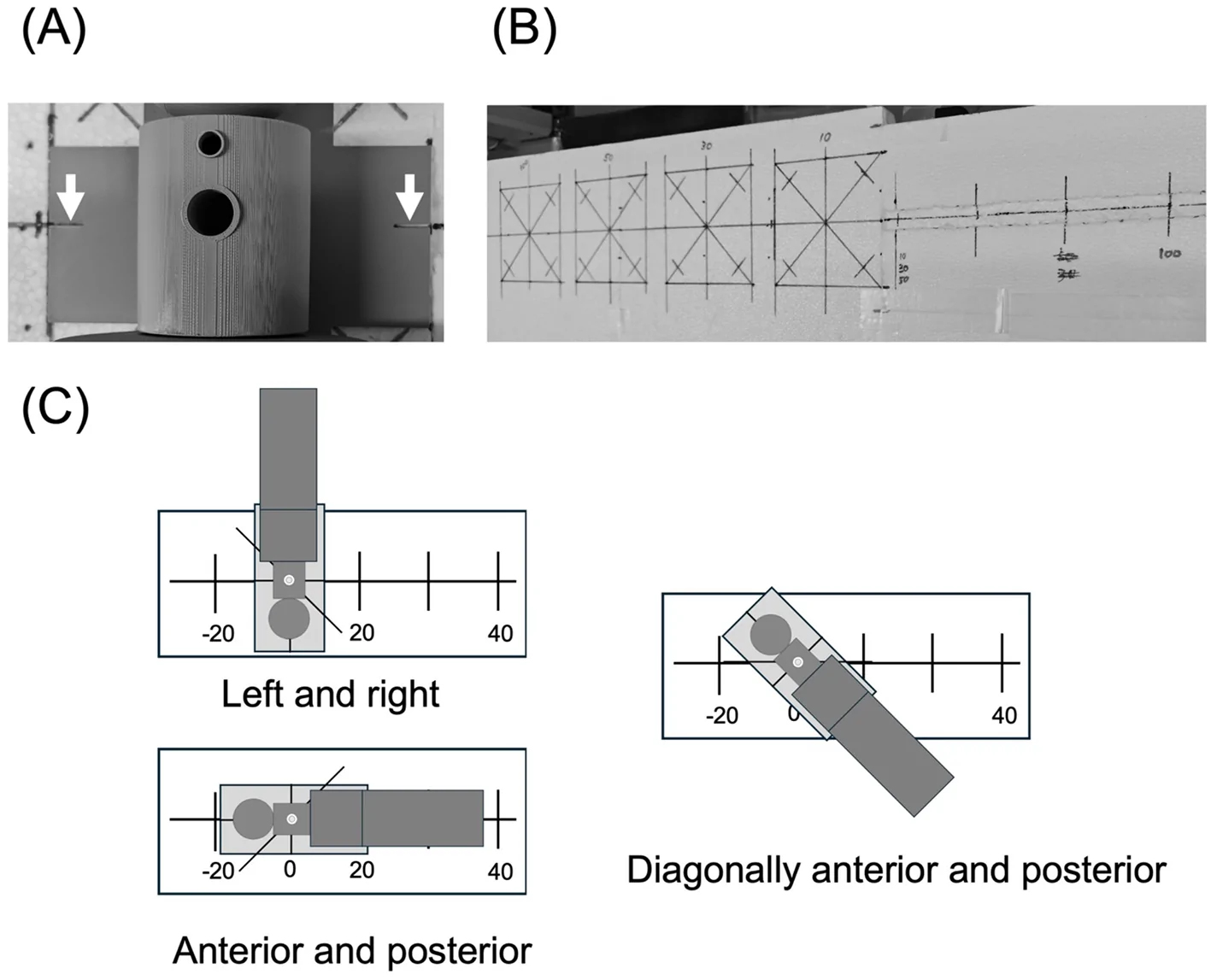
Notches **(A)**, graduated markings **(B)**, and simplified feline phantom configuration during measurement **(C)**. **(A)** Notches (arrows) were made on the auxiliary plate so that the position of the source center was consistently reproduced. **(B)** To ensure an appropriate distance between the source center and NaI(Tl) scintillation survey meter, e.g., 10, 30, 50 and 100 cm, a polystyrene support base with graduated markings was created for the entire setup. **(C)** The dose rate measurements were performed at positions anterior, posterior, left, right, and diagonally anterior/posterior relative to the source center position within the simplified feline phantom.

The ambient dose equivalent rate was measured at distances of 10 cm, 30 cm, 50 cm, and 1 m from the center of the [^2^^1^^1^At]NaAt source within the spherical insert, with an additional measurement at the simplified feline phantom surface (approximately 5 cm from the source center) performed only at the left and right lateral positions. The number of measurement points varied from two to five depending on the direction, as some close-range measurements were omitted due to physical interference by the simplified feline phantom's body (e.g., in the posterior position). To determine the relative positioning between the radiation source center and NaI(Tl) scintillation survey meter, the measurement geometry described below was constructed. To prevent rolling, both the 3D-printed jig and simplified feline phantom were equipped with an auxiliary base plate with a thickness of 1 mm. In addition, marks or notches (Figure 2A) were made on the auxiliary plate so that the position of the source center was consistently reproduced. Furthermore, to ensure an appropriate distance between the source center and NaI(Tl) scintillation survey meter, a polystyrene support board with graduated markings was created for the entire setup (Figure 2B). During all measurements, the 3D-printed jig and simplified feline phantom were placed on a 4-cm-thick expanded polystyrene board (98% air), which was supported by a steel rack. The simplified feline phantom configuration during measurements, including those in the anterior, posterior, left, right, and diagonally anterior/posterior positions, is shown in Figure 2C. Furthermore, for a conservative evaluation, the relative reference error (RRE, ±15%) of the NaI(Tl) scintillation survey meter, TCS-1172, was used to represent the measurement uncertainty of the ambient dose equivalent rate measurements.

### 2.4 Formula for calculating the effective dose rate, Ė

The manual on the proper use of sodium astatide ([^211^At]NaAt) injections for human patients (3) provides the following equation for calculating the effective dose rate [μSv/h], *E*:


$$\begin{array}{l} Ė\;=\;A\times C\times F_{a}÷L^{2} \end{array}$$ (1)

where *A* is radioactivity in the spherical insert [MBq], *C* is the effective dose rate constant of ^211^At [μSv·m^2^·MBq^−1^·h^−1^] (0.00644), *F*_*a*_ is the effective dose transmission rate, and *L* is the distance from the radiation source to the evaluation point [m]. For a conservative radiation protection assessment, *F*_*a*_ was set to the values reported by Fukano (12), assuming attenuation of photon energy by water only. Hereafter, the results obtained from calculating the external exposure dose equivalent rate using Equation 1 are denoted as ‘FORM'.

### 2.5 Monte Carlo simulation of operational quantity, Ḣ^*^(10), and protection quantities

The ambient dose equivalent rate, Ḣ^*^(10), and the protection quantities, namely effective dose (*ED*), equivalent dose to the skin (*D*_skin_), and equivalent dose to the lens of the eye (*D*_lens_), were evaluated using the Particle and Heavy Ion Transport Code System (PHITS) (13), which is a general-purpose Monte Carlo radiation transport code. While several well-established Monte Carlo codes, including MCNP (14) and GEANT4 (15), are available and have been adopted in international dosimetric studies and ICRP publications [e.g., ICRP Publication 116 (16)], demonstrating their reliability as international standards for radiation transport calculations, PHITS was selected for this study because it is widely accepted in Japan for radiation transport and shielding analyses, including regulatory assessments submitted to the Nuclear Regulation Authority of Japan.

The ambient dose equivalent rate, Ḣ^*^(10), and the protection quantity rates were calculated as


$$\begin{array}{l} {Ḣ}_{Q}=\int \Phi \left(E\right)DCC_{Q}\left(E\right)dE \end{array}$$ (2)

where Φ(*E*) [cm^−2^·s^−1^·MeV^−1^] is the photon fluence spectrum and DCC(*E*) is the fluence-to-dose conversion coefficient [μSv·cm^2^]. *Q* denotes one of the quantities considered in this study, namely Ḣ^*^(10), *ED*, *D*_skin_, or *D*_lens_.

The PHITS code was used to simulate the transport behavior of all particles emitted from ^211^At, such as absorption and scattering in water and plastic materials. The photon fluence spectrum was scored in spherical tally regions with a radius of 1.0 cm centered at the evaluation point (5, 10, 20, 30, 40, 50, 70, 100 and 150 cm from the source). Thus, the fluence used in Equation 2 represents the local photon field at the point of interest and not the fluence within individual organs.

The scored photon fluence spectra were folded with the corresponding fluence-to-dose conversion coefficients to obtain operational and protection quantities. ICRP Publication 116 provides conversion coefficients from photon fluence to radiological protection quantities based on calculations performed with the ICRP reference male and female computational phantoms (16). Accordingly, the value of *ED* used in this study corresponds to the sex-averaged effective dose defined in ICRP Publication 116. Therefore, once the photon fluence at a given location is known, protection quantities can be estimated without explicitly transporting radiation through a human phantom in the present calculations by applying the fluence-to-dose conversion coefficients derived for the ICRP reference computational phantoms. For *ED*, the primary calculation was performed using the fluence-to-effective-dose conversion coefficients given in ICRP Publication 116. As an independent verification, organ equivalent doses were additionally calculated using the corresponding fluence-to-organ-equivalent-dose conversion coefficients, and *ED* was reconstructed by applying the tissue weighting factors recommended in ICRP Publication 103. The reconstructed *ED* values agreed with those obtained directly from the fluence-to-effective-dose conversion coefficients within the statistical uncertainty of the Monte Carlo calculations.

The dose conversion coefficients of Ḣ^*^(10) used in the PHITS calculations were obtained from the report by Sato et al. (17), whereas the conversion coefficients for *ED*, *D*_skin_, and *D*_lens_ were adopted from ICRP Publication 116 (16). For all quantities, conversion coefficients for the AP irradiation geometry were used because workers are generally positioned facing either the radiopharmaceutical source or the patient during routine clinical procedures.

The Monte Carlo simulations were performed using PHITS version 3.35. Photon, alpha particle and electron transport calculations were based on the EGS5 physics model, INCL4.6 reaction model, while particle interactions above the library energy range were treated using the default PHITS nuclear reaction models. The transport and interaction processes were simulated using the standard physics models implemented in PHITS. The energy cutoffs were set to 1 keV for particles. For PHITS simulation cases, 1.0 × 10^6^ particle histories were tracked so that the statistical uncertainties (relative error) in the calculations for the performance evaluation in a spherical insert with a jig were below 1.0%. All calculations were carried out on a personal computer (Mac mini) equipped with an M4 processor and 48 GB RAM.

**Figure 3:**
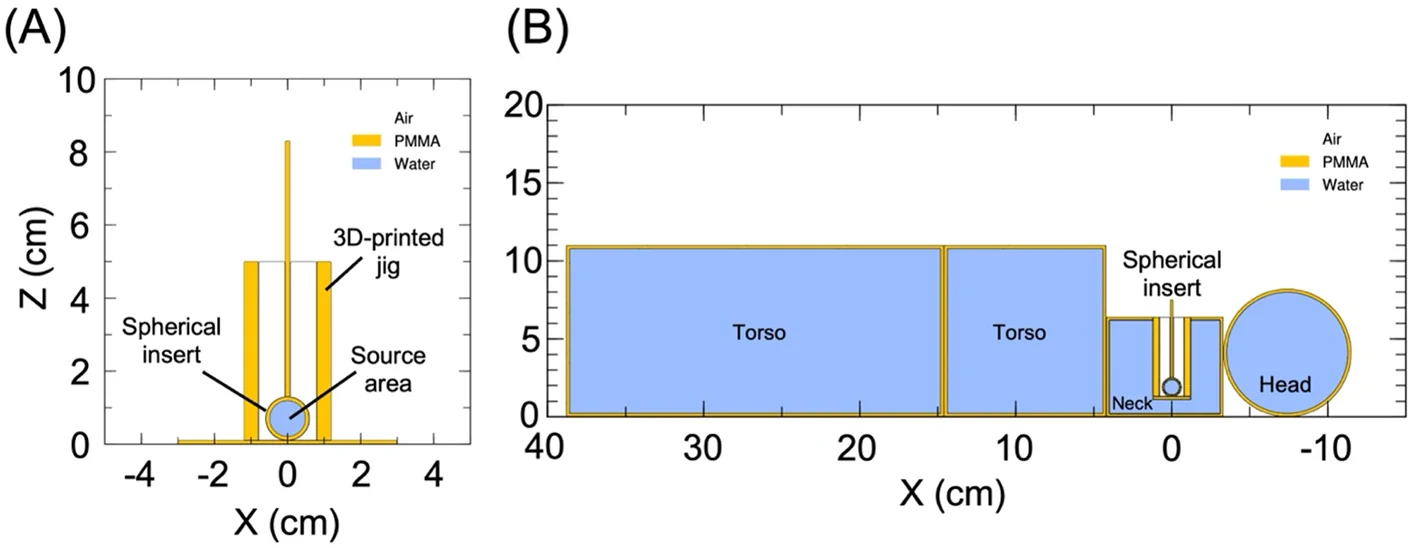
The PHITS calculation geometry of the 3D-printed jig **(A)** and simplified feline phantom **(B)**. The external dimensions were identical to those of the simplified feline phantom. The thicknesses of the spherical portion of the spherical insert and those of the simplified feline phantom (head, neck and torso) were 1 mm and 2 mm, respectively. In both geometries, a spherical insert representing the source geometry was placed, and the spherical cavity of the insert was filled with an astatine ([^211^At]NaAt) aqueous solution. The interior of the simplified feline phantom was filled with water (1.0 g/cm^3^), the 3D-printed components were filled with acrylic resin PMMA (C_8_H_2_O_5_)x, with a density of 1.18 g/cm^3^, and the remaining regions were filled with air composed of nitrogen, oxygen, and argon. Experimental setup showing the 3D-printed jig and simplified feline phantom placed on a 4-cm-thick expanded polystyrene board (98% air), which was supported by a steel rack (40 cm × 56 cm × 68 cm; width × depth × height). PHITS, Particle and Heavy Ion Transport Code System.

Figures 3A, B show the PHITS calculation geometry of the 3D-printed jig and simplified feline phantom. The external dimensions were identical to those of the simplified feline phantom described above. The thicknesses of the spherical portion of the spherical insert and those of the simplified feline phantom (head, neck and torso) were 1 mm and 2 mm, respectively. This thickness is comparable to the combined thickness of the feline epidermis and dermis (18). In both geometries, a spherical insert representing the source geometry was placed, and the spherical cavity of the insert was filled with an astatine ([^211^At]NaAt) aqueous solution. For calculations based on the simplified clinical scenario, all water regions of the simplified feline phantom were defined as the source area. The interior of the simplified feline phantom was filled with water (1.0 g/cm^3^), the 3D-printed components were filled with acrylic resin PMMA (C_8_H_2_O_5_)x, with a density of 1.18 g/cm^3^, and the remaining regions were filled with air composed of nitrogen, oxygen, and argon. For the PHITS simulations used to compare with the experimental measurements, a steel plate with dimensions of 40 cm × 56 cm and a thickness of 1 mm was placed 4 cm below the 3D-printed jig and simplified feline phantom. The expanded polystyrene board was not explicitly modeled because it consisted of approximately 98% air and was therefore assumed to be air.

### 2.6 Simplified clinical scenario

We attempted to evaluate radiation exposure to veterinary staff during [^211^At]NaAt treatment using a simplified clinical scenario (Table 1). The scenario involved transporting the radiopharmaceutical to the radioactive material storage room, compounding it in the radiopharmaceutical preparation room, administering it to the feline patient in the treatment room, and subsequently observing the feline in the animal holding room before leaving the facility. This procedure was carried out by three veterinary staff members: the veterinary administration (Vet. Admin.), veterinary restraint (Vet. Restraint), and veterinary assistant (Vet. Asst.). For each process, the specific tasks as well as the required working time and distance were defined as shown in Table 1. Effective dose rates (**Ė**) and protection quantities (*ED*, *D*_skin_, and *D*_lens_) were calculated at representative working distances using FORM and PHITS simulations, respectively. Because the FORM calculation assumes a point source, the resulting **Ė** values were presented as reference protection quantities for comparison with the operational quantities. For estimation of occupational exposure in the clinical scenario, operational quantities calculated for the spherical insert with jig were applied to tasks involving radiopharmaceutical handling before administration, whereas operational quantities calculated using the simplified feline phantom were applied to tasks performed after administration to the feline patient. Since radiopharmaceuticals are typically stored and handled within shielded containers before administration, and radioactive decay during the entire procedural sequence was neglected, the resulting dose estimates are expected to be conservative (*i.e*., to overestimate the actual occupational exposure). Per-treatment and annual occupational doses were subsequently estimated using the operational quantities calculated by PHITS, together with the task-specific working times and source-to-worker distances defined in Table 1.

**Table 1:** A simplified clinical scenario to veterinary medical staff during [^2^^1^^1^At]NaAt treatment.

Work site	Work activities	Worker^*1^	Time (min)	Distance (m)
Radioactive material storage room	Radiopharmaceutical arrival at the facility	Vet. (Admin.)	5	0.3
Radiopharmaceutical preparation room	Radiopharmaceutical preparation	Vet. (Admin.)	5	0.3
Treatment room	Animal handling and preparation	Vet. (Restraint)	10	0.3
	Radiopharmaceutical transportation	Vet. (Admin.)	1	0.3
	Radiopharmaceutical administration	Vet. (Admin.), Vet. (Restraint)	1	0.3
Animal holding room	Animal transfer	Vet. (Restraint)	1	0.5
	Animal feeding and watering	Vet. Asst	5	0.5
	Animal observation	Vet. Asst	10	1.5
	Animal exit	Vet. (Admin.) Vet. Asst	3	0.3

^*1^ Vet. (admin.) is the veterinary administration. Vet. Restraint. is the veterinary restraint. Vet. Asst. is the veterinary assistant.

## 3 Results

### 3.1 Performance evaluation in a spherical insert with a jig

**Figure 4:**
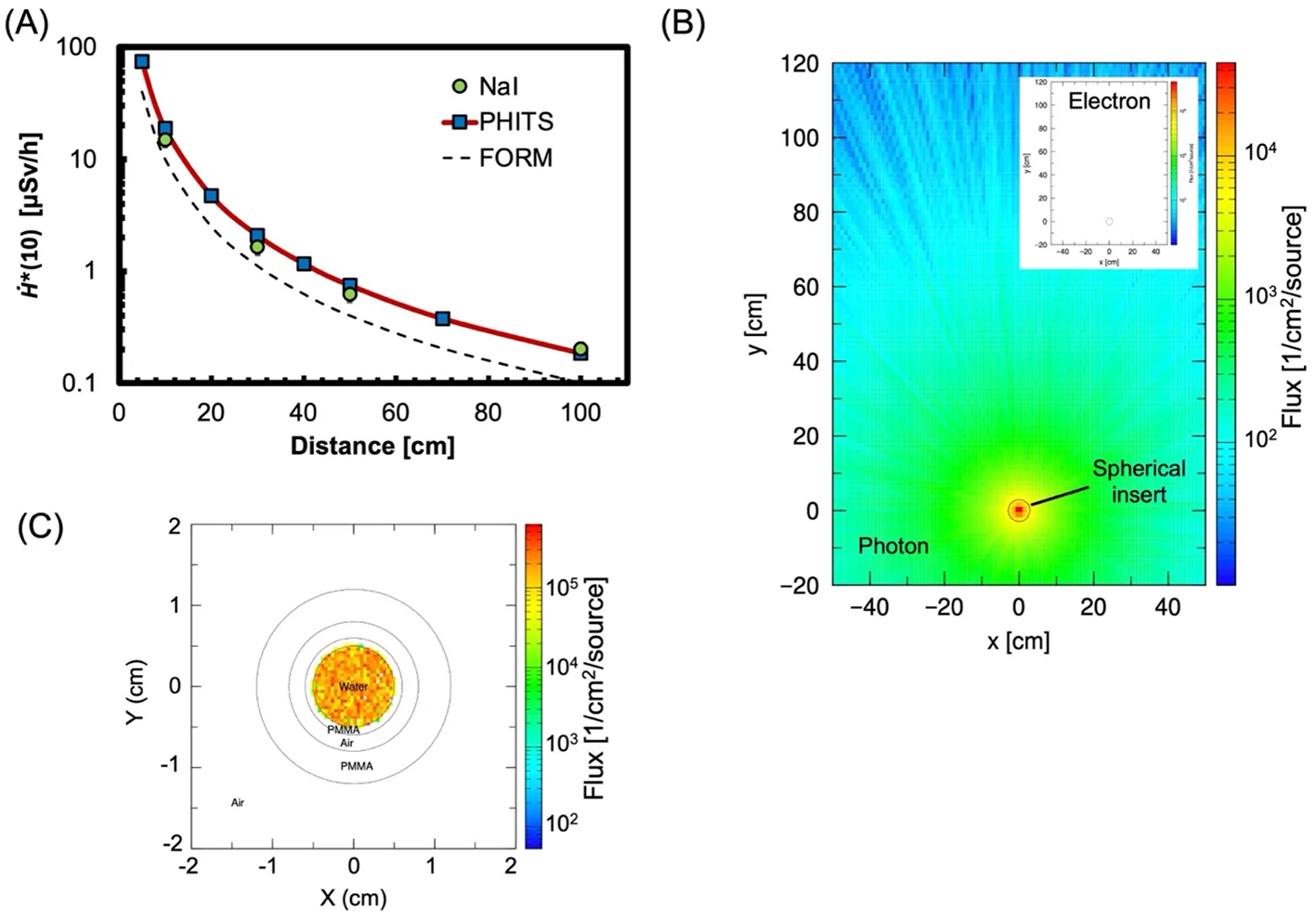
Comparison of the ambient dose equivalent rate [Ḣ*(10)] between the measurement results obtained using the survey meter and PHITS simulations **(A)**. The dashed line indicates the **Ė** values calculated using FORM. The administered activity of [^2^^1^^1^At]NaAt was 17.2 MBq. Error bars for the measured data indicate the relative reference error (RRE, ± 15%) of the survey meter. Those for the PHITS simulations represent the statistical uncertainties (relative errors) of the Monte Carlo calculations. In this experiment, the largest standard deviation (*n* = 3) of measurement was 0.86%, which was observed for the measurement at 10 cm. **(B)** PHITS-calculated photon transport tracks (fluence); the inset shows electron transport tracks. **(C)** PHITS-calculated alpha-particle tracks in the vicinity of the spherical insert. PHITS, Particle and Heavy Ion Transport Code System.

To evaluate the simulation performance of PHITS for the ambient dose equivalent rate Ḣ^*^(10), an experiment was conducted using a NaI(Tl) scintillation survey meter, in which [^2^^1^^1^At]NaAt was sealed within a spherical insert fixed by a jig. The measurement results obtained using the survey meter, PHITS simulations, and values calculated using FORM are shown in Figure 4A. The Ḣ^*^(10) showed good agreement between the survey meter measurements and PHITS simulations. The fact that the **Ė** value calculated using FORM was smaller than Ḣ^*^(10) also indicated that Ḣ^*^(10) was evaluated conservatively. These results suggest that the experimental setup using a 3D-printed jig to fix the spherical insert is appropriate for comparing the relationship between measurements and simulation results of Ḣ^*^(10) and calculated **Ė** values at distances within 1 m. To further understand the radiation components contributing to the ambient dose equivalent rate, representative particle transport tracks calculated by PHITS are shown in Figures 4B, C. Photon transport was observed outside the spherical insert and jig, whereas electron transport was confined to a relatively limited region near the source (Figure 4B). In contrast, alpha particles were completely absorbed within the 1-mm-thick spherical insert owing to their short range in matter and did not reach the exterior of the insert (Figure 4C).

### 3.2 Measurement and simulation of Ḣ^*^(10) using a simplified feline phantom

**Figure 5:**
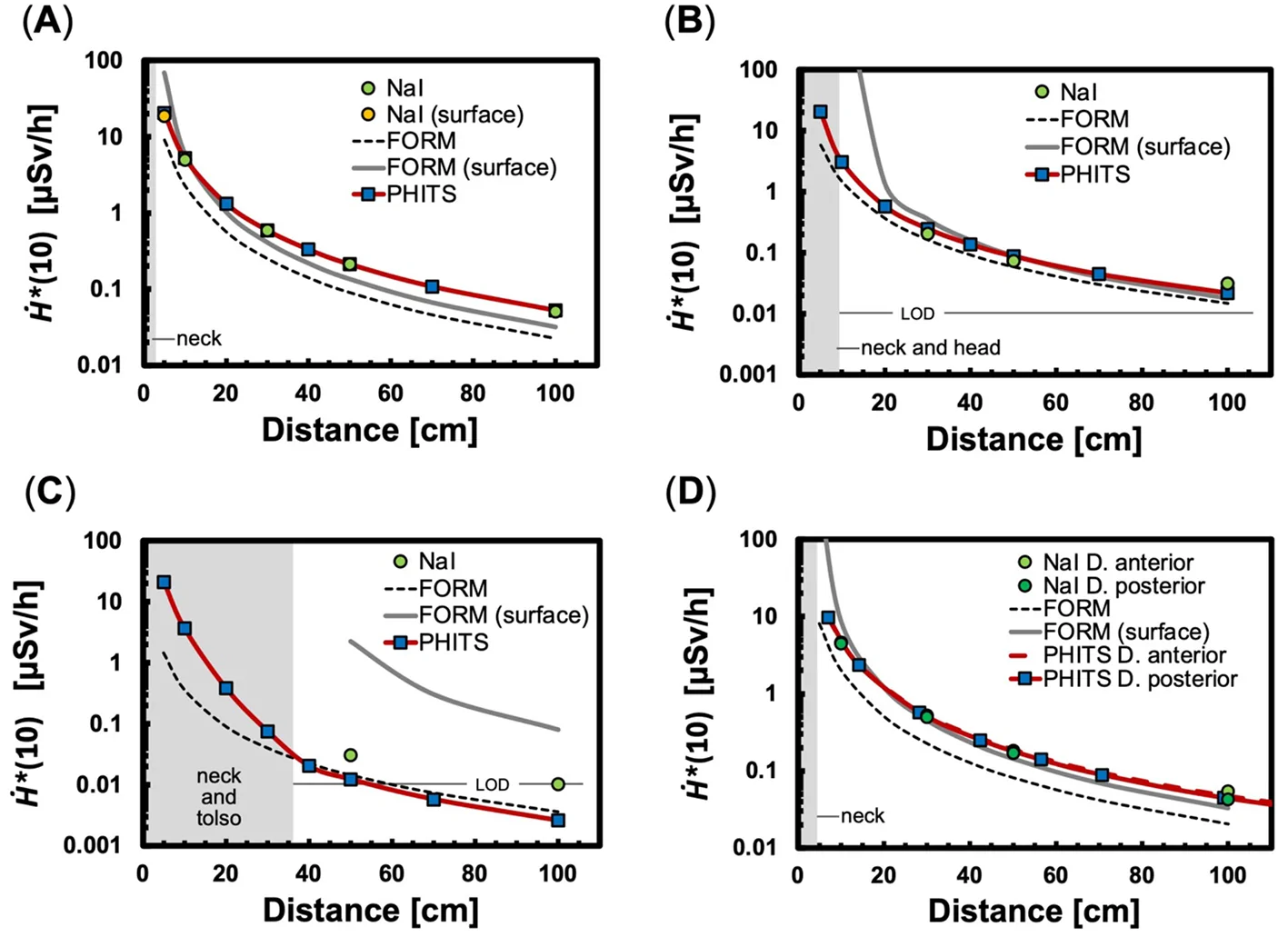
Comparison of measured ambient dose equivalent rates, Ḣ*(10), PHITS simulation results, and effective dose rate estimates (Ė) calculated using FORM in different measurement positions around the simplified feline phantom: left and right **(A)**, anterior **(B)**, posterior **(C)**, and diagonal anterior/posterior **(D)**. NaI indicates the measurement results obtained using the NaI(Tl) scintillation survey meter, TCS-1172. Error bars for the measured data indicate the relative reference error (RRE, ± 15%) of the survey meter. Those for the PHITS simulations represent the statistical uncertainties (relative errors) of the Monte Carlo calculations. The gray-shaded region indicates the simplified feline phantom. LOD is the limit of detection. NaI (surface) indicates the measurement at the body surface (at a distance of 2 cm corresponding to the reference position of the NaI(Tl) scintillation survey meter, TCS-1172). FORM (surface) shows the calculated results for **(E)** with the radiation source placed on the body surface. PHITS, Particle and Heavy Ion Transport Code System

To develop a methodology for evaluating external radiation exposure in veterinary medicine, Ḣ^*^(10) measurements of [^2^^1^^1^At]NaAt were performed using the 3D-printed simplified feline phantom and compared with Ḣ^*^(10) results obtained from numerical simulations using PHITS and **Ė** values calculated using FORM. Figure 5 shows the measurement results obtained using the survey meter (TCS-1172), PHITS simulations, and **Ė** values calculated using FORM in the left and right (Figure 5A), anterior (Figure 5B), posterior (Figure 5C), and diagonally anterior/posterior (Figure 5D) positions. The measured dose rates, Ḣ^*^(10), on the left and right sides of the neck, where the thyroid—an organ in which [^2^^1^^1^At]NaAt accumulates and which is most important for occupational exposure of veterinary staff—is located, showed good agreement with the PHITS simulation results, as observed in the performance evaluation (Figure 5A). These values were conservative compared with the **Ė** estimates obtained using FORM. In addition, the **Ė** values calculated with the radiation source placed on the body surface (FORM (surface)) overestimated the measured dose rates, Ḣ^*^(10), by a factor of 3.4 at 5 cm. In the anterior measurement position (Figure 5B), the measured dose rates, Ḣ^*^(10), showed good agreement with the PHITS simulation results. In the anterior position, FORM (surface) overestimated the PHITS simulated dose rates, Ḣ^*^(10), by a factor of 1,200 at 10 cm. In the posterior position (Figure 5C), the measured Ḣ^*^(10) values were at the limit of detection (LOD) level because of the shielding effect of the torso phantom. The background dose rate was 0.04 μSv/h. The PHITS simulation results yielded the lowest estimated values outside the phantom. By contrast, FORM (surface) overestimated the measured Ḣ^*^(10) values by two orders of magnitude. For the diagonal anterior and posterior measurement positions (Figure 5D), the measured dose rates, Ḣ^*^(10), showed good agreement with the PHITS simulation results. These values were conservative compared with the **Ė** estimates obtained using FORM. In these directions, FORM (surface) overestimated the PHITS simulated dose rates, Ḣ^*^(10), by a factor of 39 at 5 cm. Error bars representing the statistical uncertainties of the PHITS calculations are shown in Figures 5. The relative errors were generally small; however, in the posterior direction beyond 20 cm (Figure 5C), relative errors of 6%−16% were observed. This region also exhibited discrepancies between the measured and simulated values. By comparison, the relative error at 100 cm in the anterior direction was 2% (Figure 5B). These results demonstrated that the PHITS simulations at close distances within 1 m can accurately reproduce the Ḣ^*^(10) measurements of [^2^^1^^1^At]NaAt using the 3D-printed simplified feline phantom for dose rates greater than approximately 0.08 μSv/h, which is twice the background level. In addition, both the measured and simulated values were conservative with respect to the protection quantities, **Ė**. However, when the shielding effect was significant and the dose rates were close to the background level or detection limit, PHITS was unable to reproduce the measured values. Furthermore, FORM (surface) excessively overestimated the dose rates at distances within 10 cm from the body surface.

### 3.3 Gamma-ray spectrum measurement of [^211^At]NaAt

**Figure 6:**
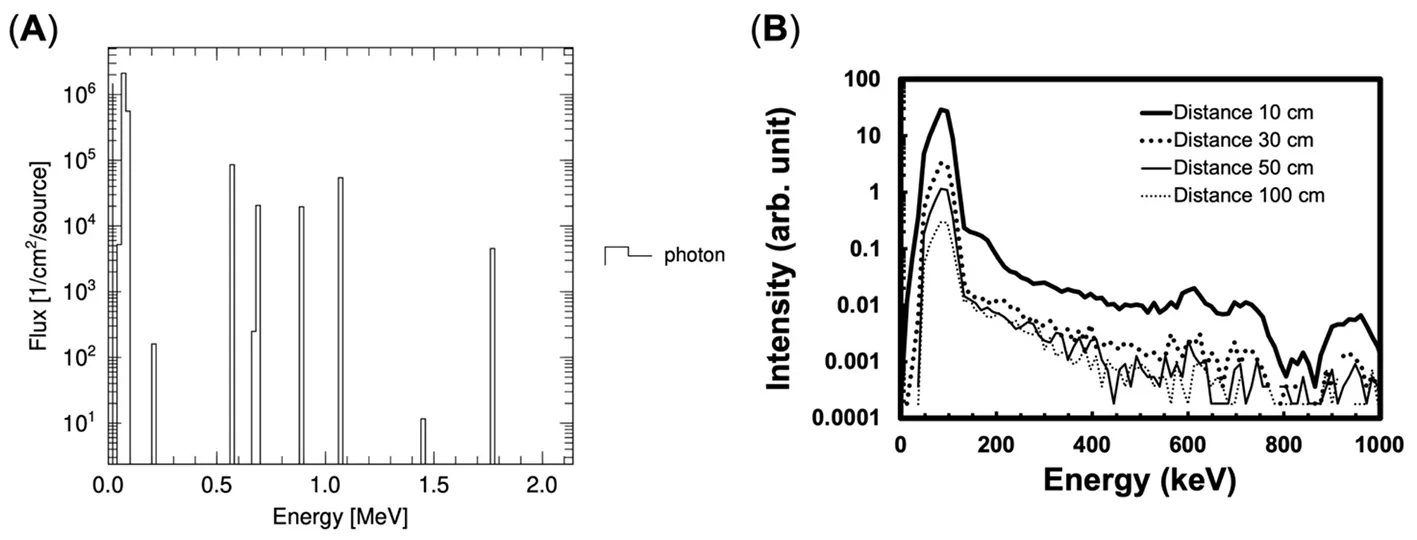
Gamma-ray spectra associated with [^2^^1^^1^At]NaAt in the simplified feline phantom. **(A)** Simulated gamma-ray energy spectra of ^211^At, ^211^Po, and ^207^Bi generated using the radioisotope source function in PHITS. **(B)** Measured gamma-ray spectra of [^2^^1^^1^At]NaAt positioned on the left (or right) side of the neck adjacent to the thyroid, where the dose rate was expected to be maximal. Spectral measurements were performed for 1 min at each distance. The spectra were normalized to the total counts obtained at a source-to-detector distance of 10 cm.

As shown in Figure 6A, using the radioisotope source function in PHITS, gamma rays emitted from ^211^At at energies of 670, 687, and 743 keV; from ^211^Po at 328, 570, and 898 keV; and from ^207^Bi at 0.33, 0.57, 0.90, 1.06, 1.44, and 1.77 MeV were simulated (13). In cases in which high levels of ^211^At and its dauters are administered, shielding of the emitted gamma rays may be required to ensure radiation protection for veterinary staff. We measured the gamma-ray spectrum of [^2^^1^^1^At]NaAt on the left (or right) side of the neck adjacent to the thyroid, where the dose rate is expected to be maximal. Figure 6B shows the gamma-ray spectrum normalized to the total counts obtained at 10 cm. Based on the measured gamma-ray spectra, gamma-rays from ^2^^1^^1^At and its progeny were detectable at 10 cm, whereas at distances greater than 30 cm, the intensities were more than one order of magnitude lower, making peak identification difficult. Considering a water-equivalent thickness of 2.9 cm at the neck position and corresponding transmission of 0.74 (12), the peak that was observed around 600 keV in the measured gamma-ray spectra was considered to originate from the 898 keV photon emitted in a transition following the decay of ^2^^1^^1^Po. These results suggest the necessity of lead shielding at close distances within 10 cm when radioactivity levels are high.

### 3.4 Radiation exposure to veterinary staff during [^211^At]NaAt treatment using a simplified clinical scenario

The occupational radiation exposure of veterinary staff was estimated using the simplified clinical scenario shown in Table 1. The calculated protection and operational quantities at representative working distances are summarized in Table 2. For these exposure conditions, *ED* values obtained directly from the fluence-to-effective-dose conversion coefficients were independently verified by reconstruction from organ equivalent doses and ICRP 103 tissue weighting factors. Agreement between the directly calculated *ED* values and the reconstructed *ED* values was confirmed within the Monte Carlo statistical uncertainties (Table 3). The estimated protection quantities per treatment and per year for each worker category are presented in Table 4. For a single treatment, the effective dose, *ED*, was estimated to be 0.91 μSv for Vet. (Admin.), 0.72 μSv for Vet. (Restraint), and 0.27 μSv for Vet. Asst. The corresponding equivalent dose to the skin, *D*_skin_, was 0.87, 0.70, and 0.24 μSv, respectively, while the corresponding eye lens dose equivalents, *D*_lens_, were 1.11, 0.89, and 0.31 μSv, respectively. Assuming treatment of four felines per day on two clinic days per week for 50 weeks per year, the annual *ED* values were estimated to be 0.37 mSv for Vet. (Admin.), 0.29 mSv for Vet. (Restraint), and 0.11 mSv for Vet. Asst. The corresponding annual *D*_skin_ values were 0.35, 0.28, and 0.09 mSv, respectively, and the annual *D*_lens_ values were 0.45, 0.36, and 0.13 mSv, respectively. All estimated annual protection quantities were substantially lower than the corresponding occupational dose limits recommended by the ICRP (19) (Table 4).

**Table 2:** Protection quantity rates calculated for a [^211^At]NaAt source using FORM and PHITS with a spherical insert and a simplified feline phantom.

Distance (m)	Protection quantity (μSv/h) ^*1^	Protection quantities simulated by PHITS in a spherical insert with a jig (μSv/h) ^*2^			Protection quantities simulated by PHITS using a simplified feline phantom (μSv/h) ^*3^		
	*Ė*	$\dot{ED}$	*Ḋ*_*skin*_	*Ḋ*_*lens*_	$\dot{ED}$	*Ḋ*_*skin*_	*Ḋ*_*lens*_
0.3	3.01	3.83	3.73	4.72	2.95	2.55	3.38
0.5	1.08	1.38	1.33	1.69	1.22	1.05	1.40
1.0	0.27	0.34	0.33	0.42	0.33	0.29	0.38
1.5	0.12	0.15	0.14	0.18	0.15	0.13	0.17

^*1^Effective dose rates (***E***) for an administered activity of 42 MBq per feline (4.2 kg × 10 MBq/kg) were calculated using the FORM assuming a point source. ^*2^Dose rates corresponding to an administered activity of 42 MBq were obtained using PHITS simulations with a spherical insert and jig. The steel plate beneath the experimental setup was excluded from the simulations, as it is not present under actual clinical conditions. ^*3^Dose rates were obtained from PHITS simulations using a simplified feline phantom. A dose of 42 MBq was administered, with 30% assumed to accumulate in the thyroid (spherical insert), while the remaining 70% was assumed to be uniformly distributed within the water regions of the head, neck, and torso phantoms excluding the thyroid. The steel plate beneath the experimental setup was excluded from the simulations, as it is not present under actual clinical conditions. PHITS, Particle and Heavy Ion Transport Code System

**Table 3:** Simulated organ equivalent doses and reconstructed effective doses based on ICRP Publication 116 organ dose conversion coefficients and ICRP Publication 103 tissue weighting factors^*1^.

Organ	Simulated equivalent dose in a spherical insert with a jig (μSv)		Simulated equivalent dose in a simplified feline phantom (μSv)	
	Female	Male	Female	Male
Brain	2.30	2.22	1.73	1.67
Breast	4.43	4.51	3.31	3.31
Colon	4.15	3.79	3.24	2.95
Bone surface	3.89	3.67	3.07	2.88
Liver	3.77	3.38	2.93	2.61
Lung	3.41	3.48	2.64	2.69
Esophagus	3.87	3.27	3.00	2.51
Ovaries	3.25		2.48	
Red bone marrow	3.66	3.41	2.85	2.64
Remainder tissues	3.43	3.24	2.64	2.49
Salivary glands	2.93	3.11	2.24	2.39
Skin	3.76	3.69	2.57	2.53
Stomach wall	3.98	3.85	3.11	3.00
Testes		4.72		3.70
Thyroid	5.08	5.01	4.01	3.96
Urinary bladder	4.62	4.06	3.61	3.15
ED^*2^	3.85	3.81	2.97	2.93
Sex-averaged *ED*	3.83		2.95	

^*1^Doses were calculated at a distance of 0.3 m from the source center and correspond to a 1-h exposure period. Consequently, the sex-averaged effective doses expressed in μSv h^−1^ are identical to those presented in Table 2. ^*2^Effective dose was reconstructed from organ equivalent doses using the tissue weighting factors recommended in ICRP Publication 103.

**Table 4:** Estimated per-treatment and annual occupational radiation exposure of veterinary staff following [^211^At]NaAt administration.

Worker	Effective and equivalent dosefor one treatment (μSv)			Effective and equivalent dose for 1 year (mSv) ^*1^		
	*ED*	*D*_*skin*_	*D*_*lens*_	*ED*	*D*_*skin*_	*D*_*lens*_
Vet. (Admin.)	0.91	0.87	1.11	0.37	0.35	0.45
Vet. (Restraint)	0.72	0.70	0.89	0.29	0.28	0.36
Vet. Asst.	0.27	0.24	0.31	0.11	0.09	0.13
Dose limit (mSv/year)				**Whole body** ^*2^	**Skin** ^*2^	**Lens of the eye** ^*2^
				20	500	20

^*1^ Four felines are treated per day on two clinic days per week for 50 weeks per year. ^*2^The occupational dose limits are based on the recommendations of the ICRP (19): 20 mSv/year for effective dose and equivalent dose to the lens of the eye, averaged over defined periods of 5 years with no single year exceeding 50 mSv, and 500 mSv y^−1^ for equivalent dose to the skin.

## 4 Discussion

As a step toward the future clinical introduction of the ^2^^1^^1^At-labeled radiopharmaceutical, [^2^^1^^1^At]NaAt, into veterinary medicine, this study aimed to establish a practical and reproducible methodology for evaluating external radiation exposure to veterinary medical staff during routine clinical practice. To achieve this objective, measurements of ambient dose equivalent rates and radiation spectra obtained using a 3D-printed simplified feline phantom were systematically combined with numerical simulations capable of reproducing the experimental conditions. This integrated approach provides a methodological basis for assessing occupational radiation exposure in veterinary nuclear medicine settings.

The particle transport tracks shown in Figures 4B, C provide insight into the radiation components of ^211^At contributing to the measured ambient dose equivalent rate. Because alpha particles were completely absorbed within the spherical insert, their direct contribution to the external radiation field was negligible. In contrast, photons penetrated the insert and surrounding materials and constituted the predominant radiation component outside the phantom. These findings indicate that the ambient dose equivalent rates measured using the NaI(Tl) survey meter primarily reflected photon radiation. This observation supports the validity of comparing the measured values with the PHITS-calculated ambient dose equivalent rates. Because the thickness of feline skin, including the epidermis and dermis, is approximately comparable to that of the spherical insert used in this study, similar attenuation of alpha particles is expected under actual clinical conditions. Nevertheless, further studies are required to evaluate the potential transport of radiopharmaceuticals through the skin and its influence on radiation exposure in veterinary radionuclide therapy.

The systematic combination of a 3D-printed simplified feline phantom with numerical simulations offers two main advantages. First, it provides an alternative to animal experiments. This would be advantageous under the current situation in which the conduct of animal experiments has been increasingly restricted worldwide (8). Second, it addresses the limitations of the currently used calculation method based on effective dose rate constants derived from point sources placed on the body surface. As shown in Figure 5, the calculated results for Ė obtained using the radiation source placed on the body surface [FORM (surface)] substantially overestimated the measured dose rates, H^*^(10). For example, overestimations of a factor of 3.6 were observed at 5 cm on both the left and right sides of the neck (Figure 5A) and of a factor of 1,800 at 10 cm in the anterior measurement position (Figure 5B). These overestimations can be effectively mitigated using our systematic combination method. This approach is expected to be effective for assessing radiation exposure at close distances within 1 m, which are relevant in the veterinary field. The systematic combination methodology developed in this study is applicable to animals other than felines, including dogs and other small animals.

In the posterior direction of the simplified feline phantom, larger relative errors were observed in the PHITS calculations than in the anterior direction. This may reflect the increased complexity of radiation transport in the posterior region, where the contribution of scattered photons is expected to be greater. In addition, variations in the background radiation level, which were not included in the simulations, may have affected the NaI(Tl) survey meter measurements. Moreover, the measured dose rates in this region were close to the detection limit of the instrument (LOD), potentially reducing the reliability of the comparison between measurements and simulations. Furthermore, the PHITS simulations conducted in this study indicated that the presence of the steel rack increased the dose rate by several percent, likely due to photon scattering and reflection. During the experiment, measurements were performed in a confined space by three to four operators who handled the phantom and survey meter. Therefore, additional photon scattering and attenuation caused by surrounding structures and personnel, including the experimenters themselves, may have influenced the measured dose rates. These environmental effects were not considered in the present simulation model and should be investigated in future studies. Therefore, the discrepancies observed in the posterior direction are likely attributable to a combination of simulation uncertainty, background fluctuations, measurement limitations, and environmental factors not accounted for in the simulations.

Radiation exposure to veterinary staff has been evaluated in previous studies. During a single veterinary procedure involving the use of [^18^F]FDG in dogs, the occupational radiation dose to veterinary staff was 7.39–55.91 μSv for the staff involved in handling the radiopharmaceutical and 3.74–44.52 μSv for staff involved in inducing anesthesia. By contrast, the occupational radiation exposure associated with [^2^^1^^1^At]NaAt administration in felines was approximately one order of magnitude lower, with estimated *ED* values ranging from 0.27 to 0.91 μSv per treatment. Assuming a clinical workload of four felines treated per day on two clinic days per week for 50 weeks per year, the annual effective dose (*ED*) was conservatively estimated to be 0.37, 0.29, and 0.11 mSv for Vet. (Admin.), Vet. (Restraint), and Vet. Asst., respectively. The corresponding annual equivalent dose to the skin, *D*_skin_, values were 0.35, 0.28, and 0.09 mSv, respectively, while the annual eye lens dose equivalents, *D*_lens_, were estimated to be 0.45, 0.36, and 0.13 mSv, respectively. All estimated protection quantities were substantially below the occupational dose limits recommended by the ICRP (19), including the annual dose limit for the lens of the eye. These findings indicate that occupational radiation exposure associated with feline [^2^^1^^1^At]NaAt therapy is minimal and is unlikely to represent a significant radiological constraint in routine clinical practice. Therefore, the present results support the practical implementation of [^2^^1^^1^At]NaAt therapy for feline thyroid disease in veterinary clinics, as the expected occupational radiation exposure, including eye lens exposure, remains well within internationally recommended dose limits. Furthermore, spectral measurements suggest that when high radioactivity is required for procedures using ^211^At-labeled radiopharmaceuticals in animals other than felines (including dogs and other small animals), the use of shielding, such as lead aprons and leaded eyewear, may be beneficial for reducing occupational radiation exposure at close distances (approximately 10 cm). An important advantage of ^211^At is its short half-life (7.2 h), which leads to low levels of occupational radiation exposure and enables same-day outpatient treatment. Consequently, these findings may substantially promote the development and wider adoption of nuclear medicine therapies in veterinary practice.

This study has some limitations. The findings of this study are currently limited to feline thyroid diseases. However, similar analyses could be performed for other clinical conditions—for example, those involving the kidneys—by modifying the torso of the phantom to allow the placement of the radiation source within the relevant anatomical site. Therefore, further investigations covering a wider range of clinical cases are warranted in future studies. In this study, radiation dose evaluation was limited to medical staff. Therefore, when introducing [^211^At]NaAt radiopharmaceuticals, it is also necessary to assess the potential radiation exposure to pet owners following treatment, including exposure that may occur during use of public transportation and daily life at home.

## 5 Conclusion

This study demonstrates a practical approach for evaluating external radiation exposure to veterinary medical staff associated with the use of the ^2^^1^^1^At-labeled radiopharmaceutical, [^2^^1^^1^At]NaAt. Measurements using a 3D-printed feline phantom, together with numerical simulations, showed good agreement between measured and calculated dose rates, and enabled the estimation of occupational exposure under a simplified clinical scenario, supporting the feasibility of this evaluation framework for veterinary applications.

## Data Availability

The original contributions presented in the study are included in the article/supplementary material, further inquiries can be directed to the corresponding author.
